# First-principles calculations on Fe-Pt nanoclusters of various morphologies

**DOI:** 10.1038/s41598-017-11236-7

**Published:** 2017-09-05

**Authors:** Alexander Platonenko, Sergei Piskunov, Dmitry Bocharov, Yuri F. Zhukovskii, Robert A. Evarestov, Stefano Bellucci

**Affiliations:** 1 Institute of Solid State Physics, University of Latvia, Kengaraga 8, Riga, LV-1063 Latvia; 2 St. Petersburg State University, 7/9 Universitetskaya nab., 199034 St. Petersburg, Russia; 3 INFN-Laboratori Nazionali di Frascati, Via Enrico Fermi 40, I-00044 Frascati, Italy

## Abstract

Bimetallic FePt nanoparticles with *L1*_0_ structure are attracting a lot of attention due to their high magnetocrystalline anisotropy and high coercivity what makes them potential material for storage of ultra-high density magnetic data. FePt nanoclusters are considered also as nanocatalysts for growth of carbon nanotubes of different chiralities. Using the DFT-LCAO CRYSTAL14 code, we have performed large-scale spin-polarized calculations on 19 different polyhedral structures of FePt nanoparticles in order to estimate which icosahedral or *hcp*-structured morphology is the energetically more preferable. Surface energy calculations of all aforementioned nanoparticles indicate that the global minimum corresponds to the nanocluster possessing the icosahedron “onion-like” structure and Fe_43_Pt_104_ morphology where the outer layer consists of Pt atoms. The presence of the Pt-enriched layer around FePt core explains high oxidation resistance and environmental stability, both observed experimentally.

## Introduction

Magnetic nanoparticles (NPs) with sizes ranging from 2 to 20 nm represent an important class of artificial nanostructured materials. Their magnetic properties essentially depend on the NP size because the thermal energy *kT* becomes comparable to the *KV* product term, where *k T*, *K* and *V* are the Boltzmann constant, the temperature, the constant of the so-called magnetic anisotropy of the nanoparticle (NP) and its volume, respectively^1^. As a result, the magnetization of the nanocluster can randomly flip direction depending on the temperature, and thus NP can be fixed in the so-called superparamagnetic state^1–3^.

FePt bulk possesses *L1*_0_^4^ chemically ordered *P*4/*mmm* tetragonal structure or chemically disordered *A1* ($Fm\overline{3}m$) cubic structure^2, 5^. It was shown recently that the atomic ratio of Fe and Pt in Fe_*x*_Pt_1–*x*_ nanoparticles (NPs) synthesized by the sol-gel method (where *x* was changed between 0.3 and 0.8) plays an essential role for the structural and magnetic properties of these NPs^6^. FePt NPs possessing a near-stoichiometric atomic percentage of Fe and Pt belong to the important class of magnetic nanomaterials. FePt *L1*_0_ NPs have attracted considerable attention because of their extremely high magnetic anisotropy^7^ making them especially useful for practical applications in solid-state devices, *e*.*g*., in high-density magnetic recording media^8, 9^, and in biomedicine, e.g., as contrast agents for the magnetic resonance imaging^10^ or as the basis for neutron activated coating when annealing the FePt core–shell NPs for cancer treatment^11^.

Magnetic properties of NPs also allow one to use them as a catalyst for the growth of carbon nanotubes (CNTs) with predictable chiralities^12^. Chirality of an arbitrary CNT depends on the direction of an external magnetic field while diameter depends on the size of FePt NP^2^. It should be also noted that FePt nanoparticles are chemically more stable than high-magnetic nanoclusters of Co and Fe, as well as other high coercive materials like CoSm_5_ and Nd_2_Fe_14_B^2^. Synthesized *L1*_0_ FePt/MnFe_2_O_4_ core-shell nanocomposites also possess attracting magnetic properties^13^.

FePt NPs were mainly synthesized using organometallic chemistry^7, 14^ or gas-phase condensation and nucleation methods^15–17^. The latter can be applied for the synthesis of NPs with various shapes: cuboctahedrons, icosahedrons, and faceted spheres^17, 18^.

A number of different FePt NP sectioning morphologies and properties simulated using first-principles calculations are described in the literature^19–28^. Free energies of (100), (001), (110), (011) and (111) flat sections of *L1*_0_ FePt bulk were calculated to estimate surface energy anisotropy of FePt NPs^19^. Evidently, {111} facets of nanocrystal were found to be energetically the most favorable. This result is important for analysis of high-resolution transmission electron microscopy images, since FePt NPs do not show obvious surface faceting on the early stage of annealing. It means that some structural features are rather depending on the kinetic factors than on the free surface energies. In ref. 20, Gruner has shown that for *L1*_0_ ordered alloys, the two (001) surfaces perpendicular to the shortened *c*-axis are preferably covered by Pt atoms analogously to the outermost shell of Fe_144_Pt_165_ NP completely covered by Pt.

The systematic survey of the size dependence of the energetic order and magnetic properties of FePt nanoparticles of various morphologies was performed by Gruner *et.al*.^21, 22^. Low-indexed surfaces of mono-metallic Fe, Co, and Pt, as well as bimetallic, ordered FePt, CoPt, and MnPt NPs were calculated using the density-functional theory (DFT)^23^. These calculations showed that elemental, Pt-covered surfaces are preferable over Fe and Co covered and mixed surfaces of the same orientation. The first-principles DFT calculations were used to determine how Pt surface segregation (exchanging interior Pt with surface Fe atoms) would affect the magnetic properties of *L1*_0_ ordered FePt NPs^28^. In ref. 29, the structural stability and structural features of FePt NPs were studied using Monte Carlo algorithms generating models of different shapes, Fe/Pt ratios and atomic compositions. It was shown that the icosahedron possesses the best structural stability and the lowest energy in comparison with all other shapes of NPs (truncated octahedron, octahedron, decahedron, or hexahedron).

Despite these studies it is not precisely known at this moment what types of particles are the most stable and which types can be obtained experimentally. The growth mechanism of NPs is still being discussed, but the recent studies bring evidences that they exhibit shell periodicity and grow by accretion of atomic layers. Still, there are many known NPs that possess shell-core structure. For example, such a type of bimetallic NPs have been simulated recently^30^, *e*.*g*., Pd_*m*_Au_*n*_^31^. The method of topological energy expression (TOP) applied for them has enabled one to determine the most energetically stable atomic arrangements by global optimization of the mutual positions of different atoms (or chemical ordering). As in the case of FePt core-shell NPs, the energetically more preferable configurations were found to be fully covered by noble metal atoms (Au).

In order to shed more light on the NP surface structure and the mechanism of NP growth, we have performed a series of DFT calculations using the CRYSTAL14 code^32^. Using the methodology suggested in ref. 33 we have calculated the Gibbs free energy to determine the lowest-energy structure of a NP surface as well as the morphology and stoichiometry of FePt nanocluster which correspond to the thermodynamically favorable surface.

The paper is organized as follows. In the first subsection of “Methods” section the computational details of *ab initio* calculations by a linear combination of atomic orbitals (LCAO) within the DFT approach used in our modelling are given. The second subsection of “Methods” section describes different atomistic models of icosahedral and hexagonal close-packed (*hcp*) FePt NPs used in calculations. The obtained results are analyzed in the “Results” section divided into three Subsections. The “Bulk calculations” subsection contains detailed information about Fe, Pt and FePt bulk crystal structure calculations. In the “Magnetic properties of FePt nanoparticles” subsection, we analyze the magnetic structure of the considered NPs. The “Thermodynamical analysis of FePt nanoparticles structures” subsection presents the thermodynamical analysis of FePt NPs. Finally, the conclusions are given in the “Summary” section.

## Methods

### Computational details

Spin-polarized large-scale LCAO calculations on FePt NPs have been performed using the CRYSTAL14 code^32^ within the DFT-PWGGA exchange-correlation functional^34–36^. For the Fe, the all-electron atomic centered Gaussian-type function basis set (BS) has been adopted in the triple-zeta valence form^37^, while for the Pt, we have used the effective core pseudopotential BS of 311*s*-1*sp*-221*p*-41*d* ^38^, with the exponents of core and valence shells being unchanged. Unique options provided by the CRYSTAL code allowed us to perform a consistent comparison between the HF and DFT descriptions of the atomic and electronic properties of molecules and solids, including performance of the hybrid exchange-correlation functionals. With respect to the plane-waves codes the CRYSTAL code allows us to avoid artificial periodicity of repeated 3D boxes and perform simulations for stand-alone nanoparticle in the most efficient manner.

The threshold parameters of CRYSTAL code (ITOLn) for evaluation of different types of bielectronic integrals (overlap and penetration tolerances for Coulomb integrals, ITOL1 and ITOL2, overlap tolerance for exchange integrals ITOL3, as well as pseudo-overlap tolerances for exchange integral series, ITOL4 and ITOL5^32^) have been set to 6, 6, 6, 6, and 12, respectively. If the overlap between the two atomic orbitals is smaller than 10^−*ITOLn*^, the corresponding integral is truncated. Further increase of threshold parameters results in much more expensive calculations yielding only a negligible gain in the total energy (10^−7^ a.u.). Calculations are considered as converged when the total energy obtained in the self-consistent field procedure differs by less than 10^−7^ a.u. in two successive cycles. Full geometry optimization has been performed for each considered model.

### Models of nanoclusters

A number of FePt nanoclusters with different stoichiometry and atom arrangement has been set up with preserved *L1*_0_ structure. There have been icosahedra and *hcp* configurations, possessing either layered or “onion-like” structures. The latter are observed and described, *e*.*g*., in ref. 39.

We have observed that the icosahedral structures formed the so-called “magic number” clusters^22^, where the number of atoms *N* is given as a function of the number *n* of closed geometric shells

1 $$N=\frac{1}{3}\mathrm{(10}{n}^{3}+15{n}^{2}+11n+\mathrm{3),}$$

**Figure 1:**
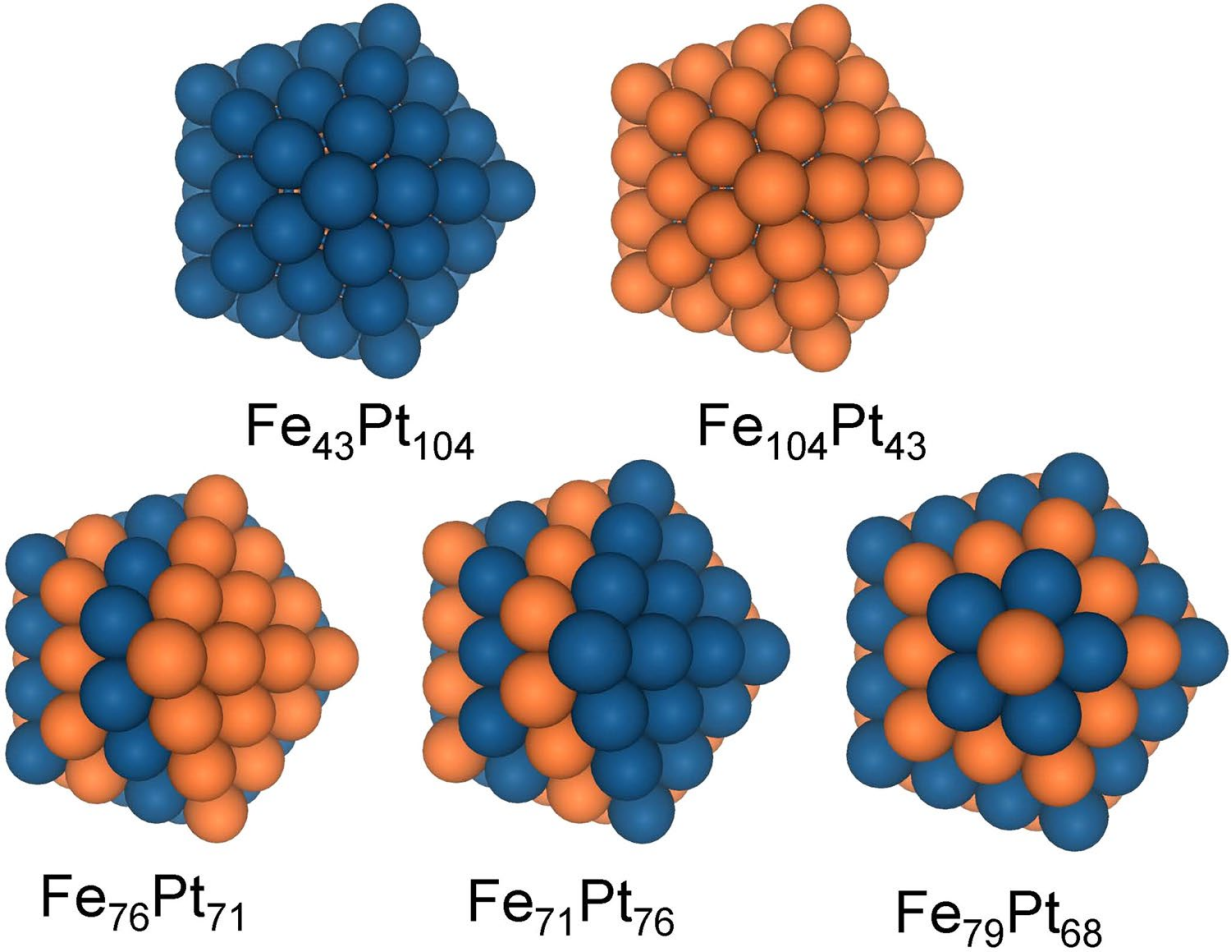
Selected icosahedral cluster models with initial morphology.

**Figure 2:**
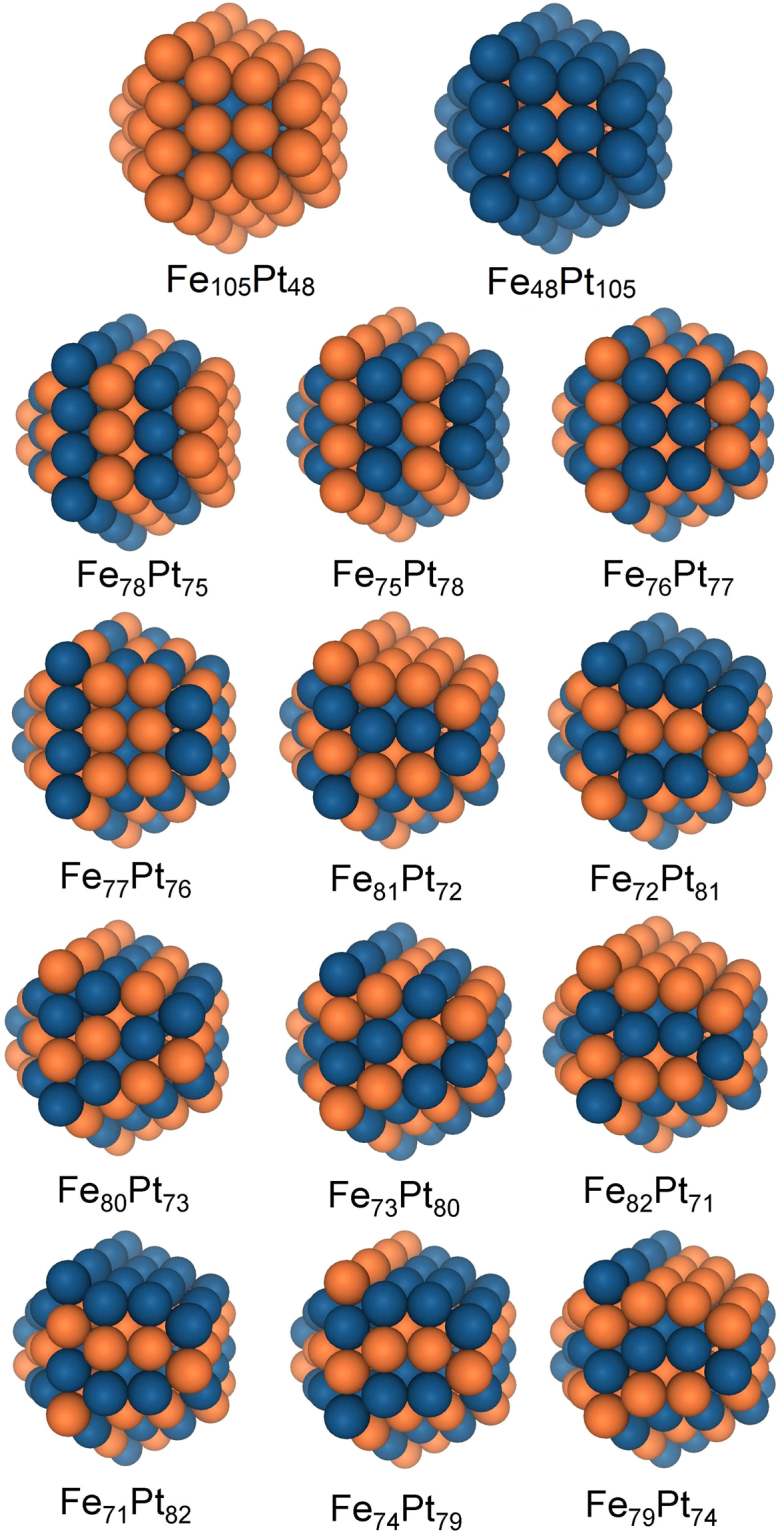
Selected *hcp* nanocluster models with initial morphology.

In this study, all chosen icosahedra were of the same size −147 atoms^40^, which corresponds to a diameter of ≈1.6 nm and number of closed atomistic shells *n* = 3 (Fig. 1). Analogous *hcp* structures consist of 153 atoms^40^, which correspond to a diameter of ≈1.4 nm (Fig. 2). In this study, we have chosen NPs of icosahedron and *hcp* structure as both contain the largest number of the most stable {111} facets. At the same time both icosahedron^17^ and *hcp* ^41^ structured FePt NPs were observed experimentally.

For both types of NPs possessing icosahedral and *hcp* configurations, we have constructed models with “onion-like” atom arrangement, which are isotropic in contrast with layered and other types of arrangements. In total, we have considered and calculated 19 NP structures. Two “onion-like” (Fe_43_Pt_104_ and Fe_104_Pt_43_), and three layered icosahedron structures (Fe_76_Pt_71_, Fe_71_Pt_76_, Fe_79_Pt_68_), each containing 147 atoms, are presented in Fig. 1.

Two “onion-like” *hcp* structures (Fe_45_Pt_108_ and Fe_108_Pt_45_), and twenty layered *hcp* structures (Fe_82_Pt_71_, Fe_71_Pt_82_, Fe_81_Pt_72_, Fe_72_Pt_81_, Fe_80_Pt_73_, Fe_73_Pt_80_, Fe_79_Pt_74_, Fe_74_Pt_79_, Fe_78_Pt_75_, Fe_75_Pt_78_, Fe_77_Pt_76_, Fe_76_Pt_77_), containing 153 atoms, are collected in Fig. 2.

## Results

### Bulk calculations

Properties of Fe, Pt and FePt 3D crystals have been calculated at the beginning in order to obtain energy reference for calculations of chemical potentials of these compounds as well as to check the quality of chosen BSs. For this purpose, we have calculated such Fe, Pt and FePt bulk properties as lattice constants, magnetic moments of atoms, and bulk moduli comparing these values with the experimental data reported in the literature.

The bulk phase of *L1*_0_ FePt has been calculated for *P*4/*mmm* space group that gives lattice constants of 2.743 Å and 3.780 Å as well as magnetic moments equal to 3.144 *μ*_B_ and 0.169 *μ*_B_ for Fe and Pt, respectively. Fe bulk has been calculated for $Im\overline{3}m$ space group yielding *a*_0_ = 2.812 Å and 2.140 *μ*_B_. For Pt bulk calculations, the $Fm\overline{3}m$ space group has been adopted that yields lattice constant of 4.029 Å. (The experimental lattice constants are equal to 2.86 Å for Fe^42^, 3.92 Å for Pt^43^, and *a* = 2.7301 Å and *c* = 3.7879 Å for FePt ordered structure^44^).

We have also calculated bulk moduli for Fe, Pt and FePt bulk, obtaining values B_0Fe_ = 220 GPa, B_0pt_ = 218 GPa and B_0FePt_ = 202 GPa, correspondingly. These results qualitatively agree with the existing experimental data: Fe bulk modulus measured at 300 K is equal to B_0Fe_ = 166.2 GPa^45^ while Pt experimental bulk modulus at 300 K is equal to B_0Pt_ = 280 GPa^46^. Finally, a bulk modulus for FePt polycrystalline *L1*_0_ structure recently measured at room temperature is equal to B_0FePt_ = 208.1 GPa^47^.

### Magnetic properties of FePt bulk and nanoparticles

**Figure 3:**
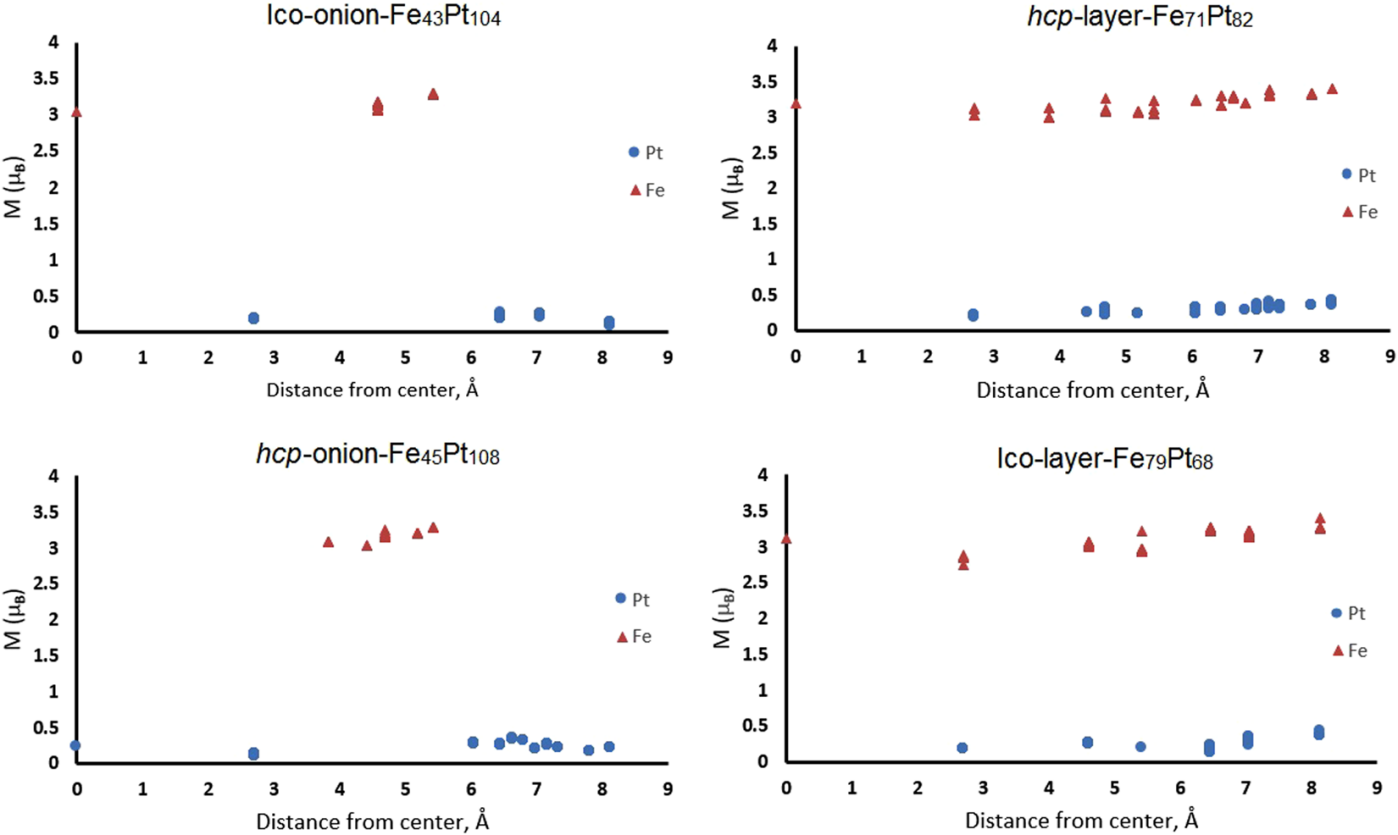
Magnetic moments of Fe and Pt atoms for some studied FePt NPs (icosahedral “onion-like” Fe_43_Pt_104_ NP, icosahedral layered Fe_79_Pt_68_ NP, *hcp* “onion-like” Fe_45_Pt_108_ NP and *hcp* layered Fe_71_Pt_82_ NP) depending on the distance between the constructed NP center and the selected atom. Values are given in *μ*_B_.

**Figure 4:**
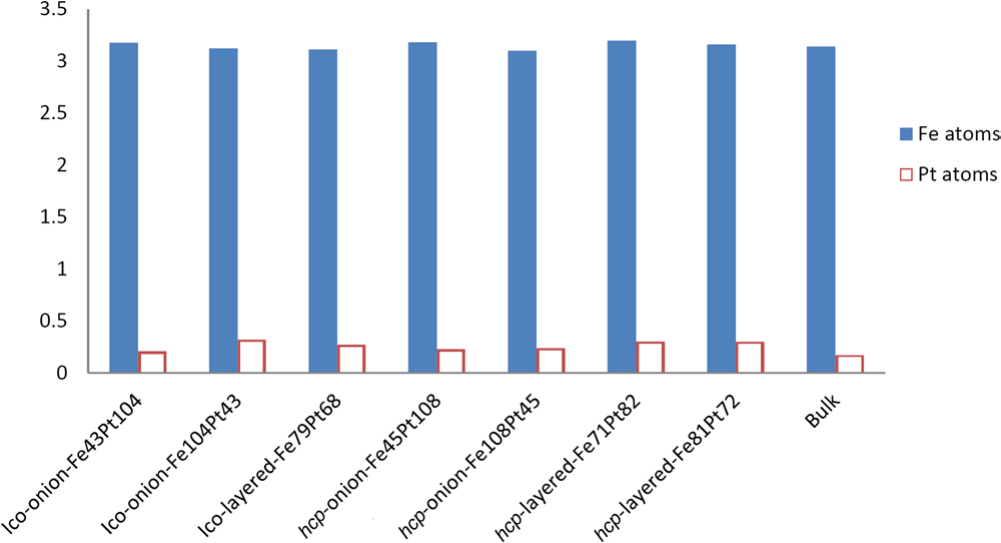
Mean magnetic moments of Fe and Pt atoms for some studied FePt NPs in *μ*_B_ (from left to right): icosahedron “onion-like” Fe_43_Pt_104_ and Fe_104_Pt_43_ and layered Fe_79_Pt_68_ structures, *hcp* “onion-like” Fe_45_Pt_108_ and Fe_108_Pt_45_, and layered Fe_71_Pt_82_ and Fe_81_Pt_72_ structures, as well as magnetic moments of Fe and Pt atoms in FePt bulk calculations.

The distribution of magnetic moments of Fe and Pt atoms has been analyzed for all considered NP structures. Magnetic moments *M*_Fe_ and *M*_Pt_ of Fe and Pt atoms inside the studied FePt NPs, which depend on the the distance between the corresponding atom and the NP center, are shown in Fig. 3 for four selected models of FePt NPs. The mean magnetic moments of Fe and Pt atoms for seven of the studied FePt NP models as well as for the FePt bulk are shown in Fig. 4. It has been found that the mean *M*_Fe_ in each NP is similar to *M*_Fe_ in bulk phase, while the mean *M*_Pt_ in NPs is about 20% higher. In all cases magnetic moments of Fe atoms grow with the distance away from the center of NP. The trend of the magnetic moment distribution for other NP models which are not shown in Figs 3–4 is similar. These values qualitatively agree with the existing experimental data for FePt NPs: ≈2.2–2.6 *μ*_B_^48–50^ for Fe atoms and ≈ 0.4 *μ*_B_ for Pt atoms^48^. Figure 3 also shows that Fe_43_Pt_104_ nanoparticle possesses high symmetry (C_2*h*_), so large numbers of Fe and Pt atoms are located at equal distance from the center.

### Thermodynamical analysis of FePt NP structures

In order to predict the most likely particle shape and composition, the detailed knowledge of the surface free energies *G* of the competing NP surface’s morphologies and internal interfaces is needed. The thermodynamic approach used in the current study in order to estimate the stability of FePt NP surfaces has been adopted from the refs 23 and 33. According to the prescription given in the ref. 33, we assume that the stable NP surface has to be in equilibrium with the FePt bulk phase. Therefore, the most stable NP has the lowest surface Gibbs free energy defined as

2 $${G}_{t}=\frac{{E}_{t}^{{\rm{FePt}}}-{N}_{{\rm{Pt}}}{\rm{\Delta }}{\mu }_{{\rm{Pt}}}-{N}_{{\rm{Fe}}}({E}_{{\rm{bulk}}}^{{\rm{FePt}}}-{\rm{\Delta }}{\mu }_{{\rm{Pt}}})}{A},$$

where *t* indicates the NP morphology (stoichiometry), *A* the NP surface area, *N*_*i*_ the number of atoms of type *i* in the NP ${E}_{t}^{{\rm{FePt}}}$ is the total energy of a NP with *t* morphology and ${E}_{{\rm{bulk}}}^{{\rm{FePt}}}$ is the FePt total energy in the *L1*_0_ bulk phase, while ${\rm{\Delta }}{\mu }_{i}={\mu }_{i}-{E}_{{\rm{bulk}}}^{i}$, (*i* = Fe, Pt) are deviations of chemical potentials for metal atoms from their energy values in the bulk phase. Since *pV* term (*V* is unit cell volume) and the differences in vibrational Gibbs free energy between the bulk solid and a corresponding NP is negligibly small^33^, we omit these two contributions. This allows us to replace the Gibbs free energies in eq. (2) by the internal energies *U* calculated from the first principles.

In order to avoid the precipitation of Fe or Pt at NP surface, as well as to prevent metal atoms from leaving the NP, the following conditions must be satisfied:

3 $$0 > {\rm{\Delta }}\,{\mu }_{{\rm{Pt}}} > {E}_{{\rm{FePt}}}^{f},$$

where ${E}_{{\rm{FePt}}}^{f}$ is the calculated formation energy of −0.48 eV for FePt bulk *vs*. −0.24 eV in the experiment^51^. Calculated formation energies for Fe and Pt bulk are −6.49 and −5.60 eV *vs*. −4.28 and −5.84 eV obtained from the experiment, respectively^51^. Negative formation energies were calculated with respect to the ground state energies of free standing atoms.

**Figure 5:**
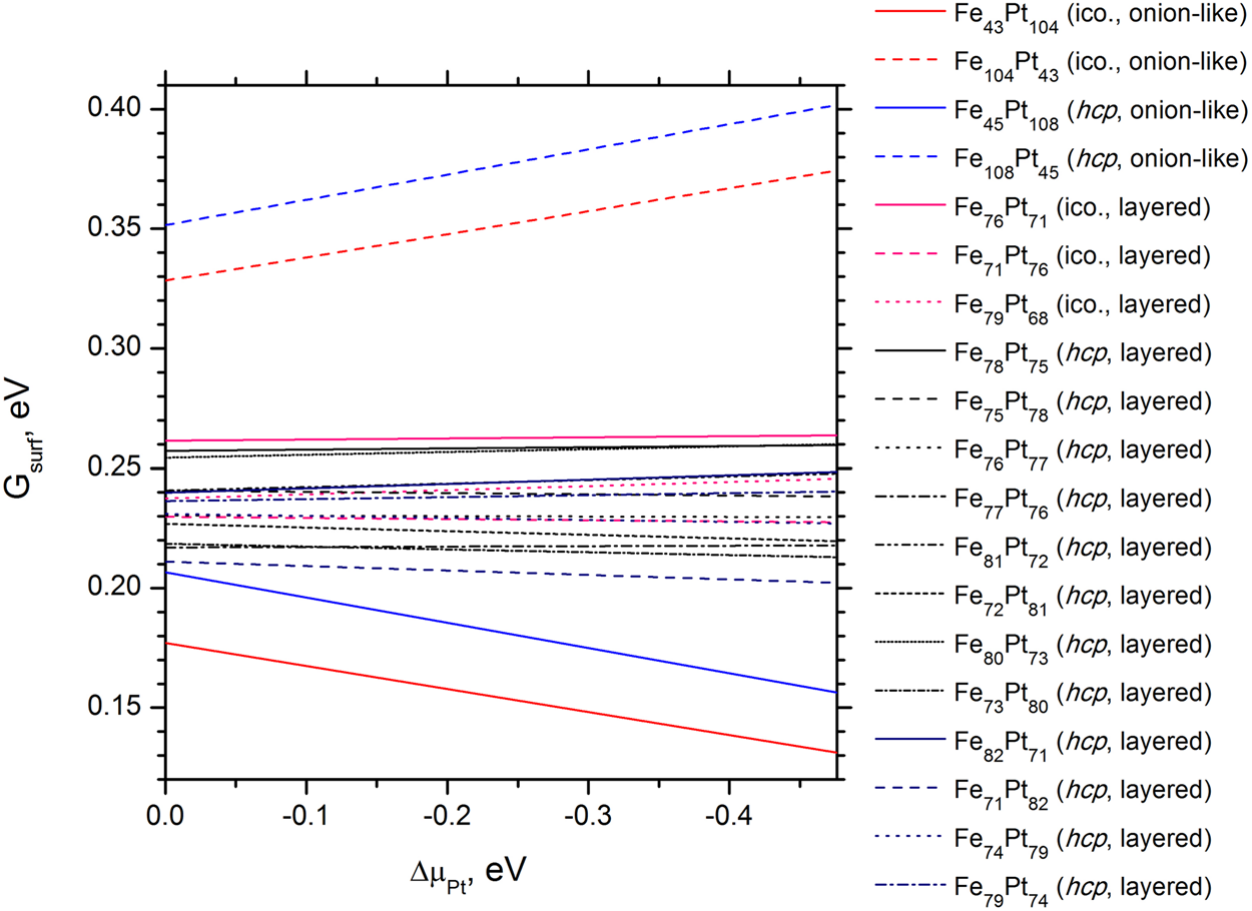
Thermodynamic stability diagram as a function of Pt chemical potential built for all FePt NPs under study. Diagram takes into account precipitation conditions for both Fe and Pt metals.

Thermodynamic stability diagram is constructed in Fig. 5 based on eqs (2 and 3). We can predict from it that the surface of Pt-covered Fe_43_Pt_104_ “onion-like” NP is the most stable, in agreement with a recent experimental observation^17^. External shells of experimentally studied FePt nanoparticles were found to be Pt-rich as energetically more stable because surface may suppress undesired magnetic exchange coupling. For more convincing conclusion on morphology of FePt NPs, we have to consider nanoparticles with diameter at least 5–6 nm, as synthesized in the aforementioned study^17^ that is beyond our current computational facilities. Nevertheless, we expect that our model of Pt-covered nanoparticle with additional 2 or 4 shells (561 and 1415 atoms, respectively) and vacancy formation at the edges of the outer shell would allow additional decrease of its free surface energy, thus leading to realistic description of magnetic FePt nanoparticles.

## Summary

In this study, we have performed large-scale DFT calculations of FePt nanoparticles of different shapes. Our calculations show that the average magnetic moment of Fe and Pt atoms does not change significantly when comparing it for bulk FePt structure and Fe_43_Pt_104_ cluster (*e*.*g*., *M*_Fe,av_ = 3.17 *μ*_B_ and *M*_Pt,av_ = 0.21 *μ*_B_ for Fe_43_Pt_104_ particle *vs*. *M*_Fe,av_ = 3.14 *μ*_B_ and *M*_Pt,av_ = 0.17 *μ*_B_ for FePt bulk phase). Using thermodynamical approach, we have found that the global minimum of surface energy corresponds to nanocluster with icosahedron “onion-like” structure and Fe_43_Pt_104_ morphology where the outer layer consists of Pt atoms only, which is in a good agreement with results obtained elsewhere^17^. This nanoparticle can be used for further simulations of enlarged cluster and adsorption of regular network of C atoms upon it resulting in a growth of carbon nanotubes.
